# A Multifaceted Approach to Problematic Smartphone Use by Adolescents in the Philippines

**DOI:** 10.1002/hsr2.71265

**Published:** 2025-09-18

**Authors:** Dalmacito A. Cordero

**Affiliations:** ^1^ Department of Theology and Religious Education (DTRE) De La Salle University Manila Philippines

## Transparency Statement

The sole author Dalmacito Cordero Jr. affirms that this manuscript is an honest, accurate, and transparent account of the study being reported; that no important aspects of the study have been omitted; and that any discrepancies from the study as planned have been explained.

Dear Editor,

I came across a recent article published in this journal regarding problematic smartphone use (PSU), a growing public health concern. The esteemed authors explored the burden of PSU, its psychological and neurobiological mediating factors, and potential prevention and intervention strategies. The findings indicated that PSU is associated with increased stress, anxiety, depression, and sleep disruptions. They concluded that PSU is a significant risk factor for mental health disturbances, particularly among adolescents and young adults [1]. With this, a multifaceted approach is needed to address the problem. I firmly share this call and want to flesh out the authors' claims by presenting the context of the Philippines specifically. In addition, I propose a combination of new media literacy and psychosocial approaches as interventions to address the public health problem.

There were 142 million cellular mobile connections that were active in the Philippines, equivalent to 122% of the total population, in the latest 2025 Global Digital Reports series. However, note that some of these connections may only include services such as voice and SMS, and some may not include access to the internet. At the start of 2025, 97.5 million individuals were using the internet, when online penetration stood at 83.8%. The Philippines has 90.8 million social media user identities in January 2025, equating to 78% of the total population of 116 million [2]. Many of these users are adolescents, either browsing the internet for learning and general information or accessing various social media platforms. The prevalent use of smartphones shows how many Filipino adolescents are dependent and even “addicted” to the digital device. Smartphone′s extended usage lessens physical activity and encourages a sedentary lifestyle that can lead to various health risks, such as chronic diseases, obesity, and mental health issues. It can also lead to a psychological condition called NOMOPHOBIA or “No Mobile Phone Phobia,” in which a person fears being detached from their mobile phone connectivity [3].

As the authors highly suggest multifaceted interventions, new media literacy (NML) is an initial intervention that adolescents and young adults must embody. NML refers to the skills and competencies required to navigate the intricacies of digital environments and various media types. It incorporates digital and media literacy components, which involve the capacity to retrieve, manipulate, and generate information within a digital environment and examine and produce media messages in many formats [4]. The role of critical thinking is enhanced in this approach. With this, the attitude of being selective and appropriate screen time is observed, given the ability to use smartphones healthily.

Aside from NML, psychosocial approaches are another intervention that must be combined with it. Since smartphone addiction is behavioral and psychological, it is appropriate to battle it according to its nature. Group cognitive behavioral therapy (CBT) is one type of this approach. It is an 8‐week intervention characterized by psychological education, group practice, sharing, and homework, consistent with the basic concepts of cognitive behavior therapy and application. This approach was utilized and conducted by six professional, master′s level students with at least a year of experience as clinical psychologists. They were supervised by a clinic director with over 10 years of experience in CBT intervention. The study resulted in a remarkable decrease in the participants' mobile phone dependence, obsessive‐compulsive symptoms, and interpersonal sensitivity [5]. Another related type under this approach is the group mindfulness‐based cognitive‐behavioral intervention (GMCI). The eight‐session intervention program is focused on cognitive reconstruction, mindfulness meditation under the framework of CBT, relaxation training, coping with relapse, activities to replace smartphone use, and setting life goals and rules. The study emphasized that GMCI could significantly alleviate smartphone addiction among university students and, thus, be an effective tool against smartphone addiction [6].

NML and psychosocial approaches are sample interventions that can be combined to create an effective strategy against PSU. It is important to note that smartphones are already part of adolescents' lives, like everyone else. It is just a matter of finding ways of using it appropriately that will not sacrifice one's health. At the same time, though smartphones have many benefits, such as bridging the gap for the sake of social relationships, real‐life connections must not be taken for granted.

## Author Contributions

**Dalmacito A. Cordero:** conceptualization; methodology; writing – review & editing; writing – original draft.

## Conflicts of Interest

The author declares no conflicts of interest.

## Data Availability

Data sharing not applicable to this article as no datasets were generated or analyzed during the current study.
